# Avian-Specific Evidence for an Estrogen Receptor Agonism Adverse Outcome Pathway Based on Chicken Embryos and LMH 3D Spheroids Exposed to Ethinylestradiol and Bisphenol A

**DOI:** 10.1021/acs.est.4c10887

**Published:** 2025-05-19

**Authors:** Tasnia Sharin, Kim L. Williams, Rudolf W. Mueller, Doug Crump, Jason M. O’Brien

**Affiliations:** † National Wildlife Research Centre, 6347 Environment and Climate Change Canada, Ottawa K1S 5R2, Canada; ‡ Department of Pathology and Laboratory Medicine, 6363 University of Ottawa, Ottawa K1H 8M5, Canada

**Keywords:** adverse outcome pathway (AOP), estrogen, vitellogenin, BPA, spheroids

## Abstract

Several adverse outcome pathways (AOPs) describe the effects of endocrine disrupting compounds on estrogen signaling. Substantial data support an AOP related to estrogen receptor (ER) antagonism, leading to decreased fecundity in fish. In this study, data were generated for an ER agonism AOP leading to reduced fecundity in avian species (AOP537). Chicken embryos and the chicken leghorn male hepatoma cell line, LMH, were used to elucidate key events associated with estrogen signaling following exposure to 17α-ethinylestradiol (EE2) and bisphenol A (BPA). Embryos were exposed via egg injection. Viability and hepatic estrogen-responsive gene expression data were collected at midincubation (embryonic day [ED] 11). Changes in plasma vitellogenin (VTG), gonad morphology and growth were evaluated prior to pipping (ED20). Both chemicals dysregulated estrogen-responsive genes in hepatic tissue and increased plasma VTG concentrations. In LMH spheroids, EE2 and BPA altered estrogen-responsive genes and VTG concentrations at 24 and 48 h, respectively. Gonadal histology revealed oocyte-type cells and loss of testicular cords in male embryos exposed to EE2 and BPA. Overall, EE2 and BPA upregulated VTG mRNA expression, increased plasma VTG, and caused impairments in gonadal development. These results contribute avian-specific evidence to support an endocrine disruption AOP describing the relationship between disrupted VTG synthesis and impaired reproduction.

## Graphical abstract

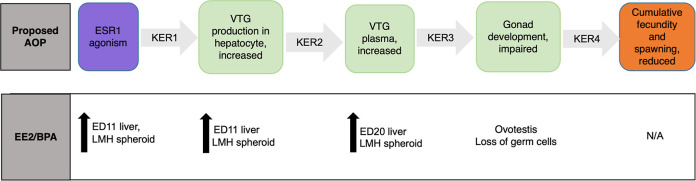

## 1 Introduction

The adverse outcome pathway (AOP) framework can be used to link molecular/cellular events to changes at the individual and/or population level.^1^ An AOP starts with a chemical–biological interaction known as a molecular initiating event (MIE), followed by a series of key events (KEs), linked through key event relationships (KERs), at different levels of biological organization, ranging from the molecular to the organismal level.^2^ Insights into mechanism of action and adverse effects of chemicals can be gained by integrating changes in gene expression with higher levels of biological organization.^3^

The endocrine system mediates biological processes, including metabolism, homeostasis, growth, and reproduction. Endocrine disrupting chemicals (EDCs) represent a diverse group of compounds that are widespread in the environment and have been detected in both humans and wildlife.^4^ In oviparous species, such as birds, the liver is an important organ in terms of the hormonal control of reproduction and it is the site of production of important yolk precursor proteins, such as vitellogenin (VTG).^5^ An AOP (AOP:30, AOP-Wiki) for the VTG pathway exists for fish and demonstrates, quantitatively, that inhibition of estrogen receptor (ER) leads to disruption of VTG synthesis, which is linked to an apical end point, reduced fecundity, and ultimately a decrease in population.^6^ High trophic level avian species in terrestrial and aquatic food webs are exposed to EDCs like 17α-ethinylestradiol (EE2) and the plasticizer, bisphenol A (BPA).^7,8^ EE2 is structurally similar to 17β-estradiol and has high affinity for ERα (ESR1) in birds.^9^ The estrogenic effects of BPA have been reported in in vivo and in vitro studies,^10–12^ and previous studies have found that BPA can bind to ER and modulate the expression of VTG in fish.^8,13^

Toxicity testing is moving from animal-based studies to faster, more ethical in vitro approaches that focus on mechanistic toxicology.^14^ Early life stage (ELS) avian toxicity testing is an alternative testing strategy that helps reduce animal use and cost. ELS is one of the most sensitive developmental periods to the toxic effects of chemicals. The avian embryo is an isolated system that can be used to determine the adverse effects of chemicals of interest and the embryonic development of laboratory model avian species such as chicken (Gallus gallus) is well-described.^15^ In vitro cell-based approaches permit dose–response evaluation and determination of mechanistic data (e.g., toxicity pathways) for chemicals while further reducing/replacing the use of animals.^16^ Furthermore, culturing cells as three-dimensional (3D) spheroids better reflects the complex in vivo microenvironment compared to 2D monolayer cell culture, which improves the relevance of in vitro testing in relation to the whole animal.^17,18^

In the current study, chicken embryos and chicken leghorn male hepatoma (LMH) cells, cultured as 3D spheroids, were exposed to a positive estrogenic control, EE2, and BPA. The objectives were to (i) generate data on different end points associated with estrogen response following exposure to EE2 and BPA in chicken embryos at two developmental stages, embryonic day (ED) 11 and 20, and (ii) expose LMH 3D spheroids to EE2 and BPA to measure molecular and biochemical effects of estrogenic compounds in vitro and compare end points between the two alternative testing strategies. The proposed AOP describes ER agonism as the MIE resulting in changes in estrogen-responsive genes, increases in plasma VTG*,* and impaired gonadal development leading to reduced cumulative fecundity and spawning (AOP537, Figure 1).

**Figure 1:**
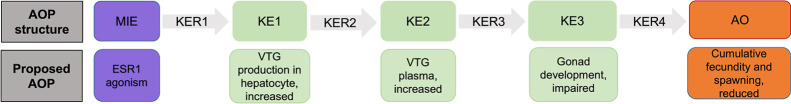
Representation of the proposed adverse outcome pathway from estrogen receptor activation leading to reduced cumulative fecundity and spawning (AOP 537).

## 2 Methods

### 2.1 Chemicals

EE2 (CAS No 57-63-6, reference standard, purity >98%) and BPA (CAS No 80-05-7, purity 97%) were purchased from MilliporeSigma (St. Louis, MO, USA). For egg injection studies, the nominal concentrations of EE2 were 0.5, 5, 25, and 50 mg/mL and 5, 10, 50, and 100 mg/mL for BPA. DMSO was the solvent control. The concentration ranges were selected based on available toxicity data from egg injection studies that used chicken and Japanese quail (Coturnix Japonica).^18,19^ For LMH spheroids, 1000 μM EE2 and BPA stock solutions were prepared in DMSO from which serial dilutions were made to yield nominal in-well concentrations ranging from 0.01 to 100 μM.

### 2.2 Egg Injection

Fertilized, unincubated chicken eggs (145 total) were purchased from the Canadian Food Inspection Agency (Ottawa, ON, Canada). Prior to incubation, eggs were weighed and candled to locate the air cell. Eggs were divided into the following groups for egg injection: DMSO (*n* = 16), EE2: 0.5 μg/g (*n* = 15), 10 μg/g (*n* = 16), 25 μg/g (*n* = 17), 50 μg/g (*n* = 17); BPA: 5 μg/g (*n* = 15), 10 μg/g (*n* = 16), 50 μg/g (*n* = 16), 100 μg/g (*n* = 17). A small hole (1–2 mm) was drilled in the center of the air cell using a Dremel tool (MultiPro, model 395, New Bern, NC, USA) through which the chemicals were injected (1 μL/g egg) using a repeater pipet. The hole was sealed with AirPoreTM Tape (Qiagen, Mississauga, ON, Canada) and eggs were left upright for 1 h at room temperature to allow the chemicals to disperse across the air cell membrane. Next, the eggs were placed in a Petersime incubator (model XI, Zulte, Belgium) and maintained at 37.5 °C and 60% relative humidity. At midincubation (ED11), eggs were candled to identify and remove any unfertilized eggs, and then, five viable eggs from DMSO control and each treatment group were randomly selected for dissection. Liver tissue (*n* = 5/group) was dissected and flash frozen in liquid nitrogen for subsequent RNA analysis. The remaining viable embryos from all treatment groups were left in the incubator until termination (ED20) as described previously.^19^ On ED20, embryos were decapitated, the yolk sac was removed, and the following measurements were recorded: embryo mass, tarsus length, head to bill length, liver mass, and gonad mass. Livers and gonads were removed for mRNA analysis and histology, respectively. Blood was collected during decapitation in heparin coated 1.5 mL tubes for genetic sex determination, and plasma was separated via centrifugation for VTG determination.

### 2.3 LMH Cell Culture

Chicken LMH cell line (ATCC CRL-2117) was purchased from Cedarlane (Burlington, ON, Canada), cultured, and maintained as previously described.^20^ All cell culture reagents were purchased from Millipore-Sigma. For spheroid culture, 14,000 cells from passage 4 were added to each well of a 96-well ultralow attachment (ULA) plate (Corning Inc., Corning, NY, USA) and grown for 4 days. The average size of a spheroid on day 5 was around 250 μm. On day 5, spheroids were treated with DMSO (0.1%), EE2 or BPA (0.1, 1,10, 100 μM) and incubated for 24 h for gene expression analysis. Next, media was aspirated, and spheroids were frozen at −80 °C for subsequent mRNA analysis. For VTG determination, spheroids were treated with DMSO, EE2, or BPA (0.1–100 μM) in phenol red free DMEM/F12 medium supplemented with 10% FBS and 5% pen/strep for 24 and 48 h.

### 2.4 Real-Time qPCR

Mid-incubation embryonic liver (∼10 mg) was homogenized using a Teflon-glass Potter-Elvehjem homogenizer (Avantor, Mississauga, ON, Canada) containing 2 mL of homogenization medium (0.25 M sucrose, 1 mM HEPES buffer, pH 7.1) on ice. RNA from male embryo livers (EE2 *n* = 4/group, BPA *n* = 5/group) and LMH spheroids (*n* = 3/group; 2 spheroids pooled per replicate) was extracted using the RNeasy Mini kit (Qiagen) and PureLink Micro RNA kit (Invitrogen, Waltham, MA, USA), respectively, following the manufacturer’s instructions. Female embryo livers were excluded from mRNA analysis due to the small sample size. RNA concentration and purity (*A*_260_/*A*_280_ = 1.9–2.1 and *A*_260_/*A*_230_ = 2.0–2.2) were determined using a NanoDrop 2000 spectrophotometer (ThermoFisher Scientific, Mississauga, ON, Canada). Approximately 100 ng of RNA from midincubation livers or LMH spheroids was reverse transcribed using the QuantiTect Reverse Transcription kit (Qiagen) according to the manufacturer’s protocol. A customized chicken PCR array comprising 9 estrogen-responsive genes, 2 housekeeping genes (EEF1A1 and β-actin), and a no template control (NTC) was used in the present study (Table S1). Real-time qPCR was performed with an RT^2^ SYBR Green ROX master mix (Qiagen) and the CFX96 Well Real-Time PCR Detection System (BioRad, Hercules, CA, USA). The thermocycle program consisted of an enzyme activation step at 95 °C for 10 min, followed by 40 cycles at 95 °C for 30 s and 60 °C for 1 min.

### 2.5 Genetic Sex Determination

Genomic DNA was isolated from 50 μL of blood from ED20 embryos. Blood samples were suspended in 100 μL of 5% Chelex 100, vortexed and incubated at 95 °C for 5 min. Avian sex determination primers for the CHD gene, 2550F/2718R (Invitrogen),^21^ were used to determine sex, and the PCR was run on a RealPlex2 thermocycler (Eppendorf, Mississauga, ON, Canada). The PCR products were run on a 3% agarose gel for 90 min to determine the number of bands; two for females, one for males.

### 2.6 Vitellogenin Assay

Plasma VTG concentration was determined in confirmed male embryos at termination (ED20) exposed to 0.5 and 25 μg/g EE2 (*n* = 4) and 5, 10, and 50 μg/g BPA (*n* = 4–5). The following dose groups were excluded due to an insufficient number of male embryos for statistical analysis: 5 and 50 μg/g of EE2 and 100 μg/g of BPA. LMH spheroids were treated with 0.1 to 100 μM EE2 and BPA for 24 and 48 h (*n* = 3/dose). After the exposure period, the spheroids were lysed via pipetting and placed on a plate shaker for 45 s, followed by centrifugation at 125 *g* for 1 min. The resulting supernatant was used to determine VTG concentration using a chicken VTG ELISA kit (Abbexa, Cambridge, UK) following the manufacturer’s instructions. The absorbance (450 nm) was read with an Infinite 200 PRO microplate reader (Tecan Life Sciences, Mannedorf, Switzerland).

### 2.7 Gonad Histology

Gonads (testes) were placed in labeled histology cassettes and fixed in 10% paraformaldehyde for 48 h and transferred to 70% ethanol for long-term storage. The samples were dehydrated in graded alcohols, cleared in xylene, and embedded in paraffin blocks for sectioning. The samples were sectioned at 5 μM thickness, affixed to glass slides, and stained with hematoxylin and eosin (Louise Pelletier Histology Core Facility, University of Ottawa). Histological sections were analyzed using a Zeiss Axioscan microscope (Toronto, ON, Canada) interfaced with a Lumenera INFINITY1–1 M digital camera and Lumenera Infinity Analyze software (Ottawa, ON, Canada).

### 2.8 Data Analysis

Real-time qPCR cycle threshold (Ct) values were normalized to the housekeeping genes, EEF1A1 and β-actin, using the 2^–ΔCt^ method.^22^ Fold change was determined relative to the DMSO control group. The fold change data were log2 transformed to account for unequal variances prior to ANOVA analysis. The resulting *p*-values between control and treatment groups were adjusted with the false discovery rate (FDR) fixed at 5% in R (Ver. 4.3.1).

VTG concentration in plasma and LMH spheroids was determined from the VTG standard curve (*R* ^2^ = 0.97). Changes in VTG concentration compared to DMSO control were determined by two-way ANOVA (chemical and dose as the two factors) followed by Bonferroni’s multiple comparisons (*p* ≤ 0.05) test in Graphpad Prism ver 6.07.

The viability of embryos at midincubation (ED11) was calculated using the log-rank Mantel–Cox test, and midincubation lethal dose (LD_50_) values of EE2 and BPA were determined from a nonlinear, variable slope (log­[inhibitor] vs normalized response) regression. Significant differences in morphometrics from DMSO control were calculated using one-way ANOVA followed by Dunnett’s multiple comparison test (*p* ≤ 0.05; Graphpad Prism ver6.07).

## 3 Results and Discussion

### 3.1 MIE: EE2 and BPA Upregulated ESR1 mRNA Expression

Many EDCs can disrupt endocrine signaling by binding to nuclear ERs, ERα and ERβ, and G protein-coupled estrogen receptor (GPER) leading to an increase in estrogen production. The main ER in hepatocytes is ERα, which is encoded by the gene, ESR1 (AOP537).^23^ Exposure to EE2 (25 μg/g) and BPA (50 μg/g) upregulated ESR1 mRNA expression by 5.78 and 2.69-log2 fold change in mid-incubation (ED11) livers. There was a concordant concentration-dependent increase in ESR1 expression (1.66 to 2.79-log2 fold change) following exposure of LMH spheroids to 1, 10, and 100 μM EE2, whereas only 100 μM BPA upregulated ESR1 expression (1.71-log2 fold change) after 24 h exposure (Table 1). Similarly, EE2 and BPA upregulated ESR1 expression in zebrafish embryos and mice,^24,25^ demonstrating concordance among species as well as between in vitro and in vivo exposures for this molecular initiating event.

**Table 1:** Changes in ESR1 mRNA Expression in Mid-Incubation Livers (ED11) and LMH Spheroids after 24 h following Exposure to 17α-Ethinylestradiol (EE2) and Bisphenol A (BPA)^a^

log2 fold change			
	dose	EE2	BPA
ED11 livers	25 μg/g	**5.78** ^b^	-^c^
	50 μg/g	-^c^	**2.69** ^b^
LMH spheroids	0.1 μM	0.96	0.11
	1 μM	**1.66** ^b^	0.21
	10 μM	**1.79** ^b^	0.33
	100 μM	**2.79** ^b^	**1.71** ^b^

a Changes in mRNA expression values are expressed as log2-fold change compared to the DMSO control. Significant fold changes are in bold (*p* ≤ 0.05).

b Represents upregulation.

c “-” not analyzed.

### 3.2 KE1: EE2 and BPA Dysregulated mRNA Expression of Estrogen-Responsive Genes in Mid-incubation Livers and LMH 3D Spheroids

Upregulation of ESR1 can activate ERα, which modulates the expression of estrogen-responsive genes in the liver involved in many physiological processes such as lipid synthesis and metabolism, bile acid homeostasis, and the thyroid hormone pathway.^23,26^ The customized PCR array included genes associated with these physiological processes; that is, estrogen signaling (apovitellenin [APOV1], vitellogenin II [VTG2]), lipid homeostasis (carnitine palmitoyltransferase I [CPT1A], cathepsin D [CTSD], cytochrome P450 family 7 subfamily A member 1 [CYP7A], liver basic fatty acid binding protein [LBFABP], stearoyl-CoA desaturase [SCD]), bile acid homeostasis (fibroblast growth factor [FGF19]), and the thyroid hormone pathway (thyroid hormone responsive spot 14 [THRSP]). LBFABP was omitted from the analysis due to late cycle threshold (Ct) values (Ct > 35) and no Cts in ED11 livers. Hepatic mRNA expression of the remaining eight estrogen-responsive genes was determined in mid-incubation embryos following exposure to 25 μg/g EE2 and 50 μg/g BPA. EE2 significantly upregulated the expression of APOV1 (17.2-log2fold), CPT1A (1.7-log2fold), SCD (6.2-log2fold), THRSP (1.6-log2fold), and VTG2 (15.2-log2fold) and downregulated the expression of CYP7A1 (−2.9-log2fold) and FGF19 (−2.7-log2fold) (Table 2). BPA significantly upregulated the expression of CTSD (1.3-log2fold), SCD (3.7-log2fold), THRSP (1.2-log2fold), and VTG2 (1.5-log2 fold) (Tables 2 and S2). Overall, there was good concordance in the direction of gene expression dysregulation between the positive control, EE2, and the putative EDC, BPA, in mid-incubation chicken liver tissue.

**Table 2:** Hepatic mRNA Expression in Mid-Incubation Chicken Embryos (ED11) following Exposure via Egg Injections to 17α-Ethinylestradiol (EE2, 25 μg/g-, *n* = 4) and Bisphenol A (BPA, 50 μg/g, *n* = 5) on ED0^a^

log2 fold change								
chemical	APOV1	CPT1A	CTSD	CYP7A1	FGF19	SCD	THRSP	VTG2
DMSO	0	0	0	0	0	0	0	0
EE2_25 μg/g	**17.2** ^b^	**1.7** ^b^	**1.9** ^b^	**–2.9** ^c^	**–2.7** ^c^	**6.2** ^b^	**1.6** ^b^	**15.2** ^b^
BPA_50 μg/g	1.8	–0.1	**1.3** ^b^	0.8	–0.6	**3.7** ^b^	**1.2** ^b^	**1.5** ^b^

a Changes in mRNA expression are expressed as log2-fold change compared to the DMSO control. Significant fold changes are indicated in bold (*p* ≤ 0.05).

b Represents upregulation.

c Represents downregulation.

LMH spheroids were treated with 0.01 to 100 μM EE2 or 0.1 to 100 μM BPA for 24 h to determine concentration-dependent changes in the expression of the same eight genes evaluated in ovo (plus LBFABP for a total of 9 genes). Concentration-dependent upregulation of APOV1 (2.5–8.3-log2FC), CPT1A (2.3–4-log2FC), SCD (2–3.4-log2FC), and VTG2 (1.3–6.9-log2FC) was observed following EE2 exposure. Expression of CTSD (2.3-log2FC) was upregulated only at 10 μM EE2, while upregulation of THRSP (3.1–3.2-log2FC) was observed at 10 and 100 μM EE2. Exposure to EE2 led to concentration-dependent downregulation of CYP7A1 (−2.0 to −4.3-log2FC), FGF19 (−2.5 to −3.4-log2FC), and LBFABP (−1.7 to −2.4-log2FC) (Table 3). Exposure to BPA resulted in concentration-dependent dysregulation of APOV1 (2.5–4.8-log2FC), CPT1A (1.6 to 3.3-log2FC), CTSD (1.9 to 3.4-log2FC), and VTG2 (2.5 to 4.4-log2FC). BPA exposure also upregulated expression of FGF19 (1.7–3.2-log2FC), SCD (2.2–2.3-log2FC), and THRSP (2.9-log2FC). Expression of LBFABP was downregulated (−2.7-log2FC) at the highest concentration of BPA. There was no change in the expression of CYP7A1 after BPA treatment (Tables 3 and S3).

**Table 3:** Changes in mRNA Expression in LMH Spheroids following Exposure to 17α-Ethinylestradiol (EE2) and Bisphenol A (BPA) for 24 h^a^

chemical	dose (μM)	APOV1	CPT1A	CTSD	CYP7A1	FGF19	LBFABP	SCD	THRSP	VTG2
EE2	0	0.0	0.0	0.0	0.0	0.0	0.0	0.0	0.0	0.0
	0.01	**2.5** ^b^	0.3	**–1.6** ^c^	–1.0	–0.7	–1.0	0.0	–0.4	**1.3** ^b^
	0.1	**4.3** ^b^	**2.3** ^b^	0.7	**–2.6** ^c^	–1.1	–0.8	**2.0** ^b^	0.4	**2.3** ^b^
	1	**5.0** ^b^	**3.0** ^b^	**–2.6** ^c^	**–2.0** ^c^	–0.7	–0.9	**2.0** ^b^	1.0	**2.9** ^b^
	10	**6.7** ^b^	**3.2** ^b^	**2.3** ^b^	**–3.2** ^c^	**–3.4** ^c^	**–1.7** ^c^	**2.8** ^b^	**3.1** ^b^	**4.5** ^b^
	100	**8.3** ^b^	**4.0** ^b^	0.7	**–4.3** ^c^	**–2.5** ^c^	**–2.4** ^c^	**3.4** ^b^	**3.2** ^b^	**6.9** ^b^
BPA	0	0.0	0.0	0.0	0.0	0.0	0.0	0.0	0.0	0.0
	0.1	0.0	–0.1	–0.4	–1.4	**–1.9** ^c^	–0.2	–0.9	–0.8	0.3
	1	**2.5** ^b^	**1.6** ^b^	–1.1	–1.3	–1.2	–1.1	0.6	–0.6	1.6
	10	**1.9** ^b^	**2.7** ^b^	**1.9** ^b^	0.3	**3.2** ^b^	–1.2	**2.3** ^b^	0.7	**2.5** ^b^
	100	**4.8** ^b^	**3.3** ^b^	**3.4** ^b^	2.0	**1.7** ^b^	**–2.7** ^c^	**2.2** ^b^	**2.9** ^b^	**4.4** ^b^

a Changes in mRNA expression are expressed as log2-fold change compared to the DMSO control (*n* = 3/dose group). Significant fold changes are in bold (*p* ≤ 0.05).

b Represents upregulation.

c Represents downregulation.

The directionality of changes in gene expression was consistent between mid-incubation livers and LMH spheroids after EE2 treatment. There were inconsistencies between the two models for APOV1, CPT1A, and FGF19 expression after BPA exposure. The three genes were unaffected in the midincubation livers, whereas in LMH spheroids they were upregulated. APOV1 and CPT1A were also upregulated in LMH spheroids following BPA exposure in a previous study^27^ demonstrating concordance among in vitro results. Developmental stage may play a role in this divergent response given that mid-incubation embryos were evaluated for the in ovo exposure, while LMH cells are derived from an adult male chicken.

Upregulation of the two markers of xenoestrogen exposure, VTG2 and APOV1, was previously observed in response to EE2 and BPA treatment in Japanese quail and zebrafish embryos.^24,28,29^ EE2 can disrupt bile acid homeostasis in rats by dysregulating the expression of CYP7A and fibroblast growth factors.^30^ EE2 induced SCD expression in the human MCF cell line,^31^ and the expression of SCD and THRSP was altered following E2 exposure in adult chicken livers.^32^ The expression of CPT and CTSD was found to be regulated by E2 in rats and the human MCF7 cell line.^33,34^ Exposure to BPA has been shown to impair metabolic pathways based on upregulation of CPT1A, SCD, FGF19, and THRSP expression in rats and mice.^35–37^ Overall, the selection of target genes for the custom array was effective in terms of screening estrogenic effects based on previous studies with multiple organisms/life stages.

### 3.3 KE2: EE2 and BPA Increased Vitellogenin Concentration in Plasma and LMH 3D Spheroids

VTG is synthesized by hepatocytes and secreted by the liver into the blood for transport to the oocytes in hens. Exposure to estrogenic compounds in immature chickens can induce VTG production in the liver. Since chicken embryos do not have developing oocytes, VTG protein accumulates in the plasma.^5^ In plasma samples from ED20 male embryos, the low dose (0.5 μg/g) and high dose (25 μg/g) of EE2 increased plasma VTG levels (Figure 2). The highest concentration of BPA (50 μg/g) also increased the VTG concentration compared to the control. Both EE2 and BPA have been found to increase plasma VTG in juvenile fish,^38,39^ demonstrating concordance across oviparous model species. VTG concentration was measured in LMH spheroids at two time points, 24 and 48 h. There was an increase in VTG protein level after 24 h of exposure to 100 μM EE2 and a concentration-dependent increase at 10 and 100 μM EE2 after 48 h in LMH spheroid lysate and medium. There was no change in VTG concentration after 24 h exposure to BPA; however, an increase was observed at 100 μM BPA at 48 h (Figure 3). The higher induction at 48 h after exposure to both chemicals suggests that changes in VTG mRNA expression occur at an earlier time point, followed by changes in protein and plasma concentration. The time- and dose-dependency of this relationship contributes a strong weight of evidence to the proposed adverse outcome pathway.

**Figure 2:**
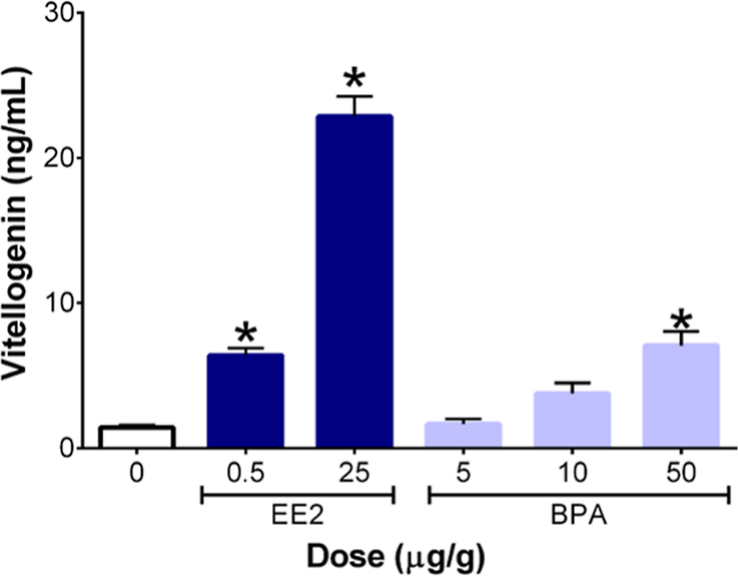
Effects of in ovo 17α-ethinylestradiol (EE2) and bisphenol A (BPA) exposure on plasma vitellogenin concentration in male ED20 embryos (*n* = 4–5). “*” represents significance compared to DMSO-treated control embryos, and error bars represent the standard error of the mean (+SEM) (*p* ≤ 0.05).

**Figure 3:**
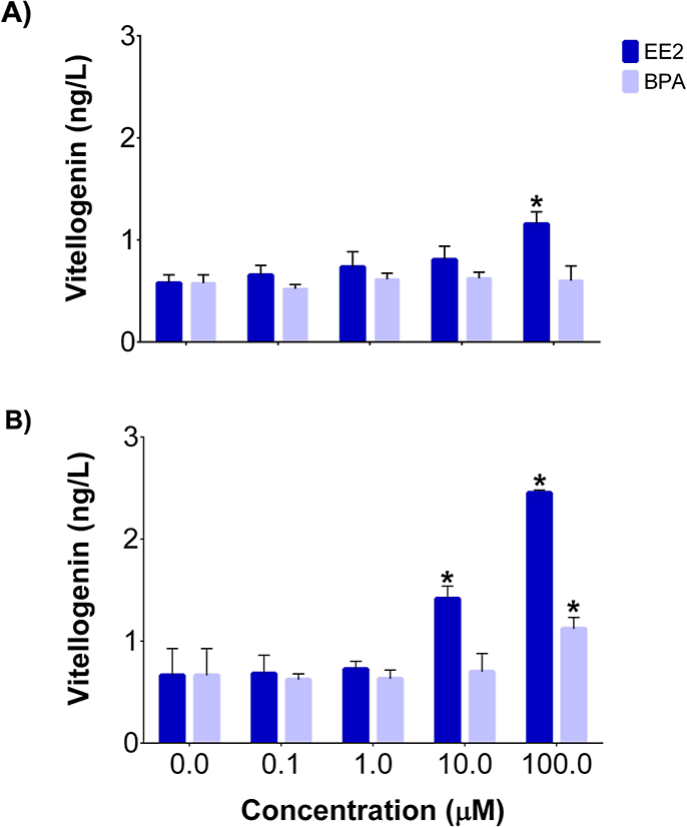
Vitellogenin concentration in LMH spheroids and culture medium after exposure to EE2 and BPA for (A) 24 h and (B) 48 h (*n* = 3). “*” represents significance compared to DMSO-treated control embryos, and error bars represent the standard error of the mean (+SEM) (*p* ≤ 0.05).

### 3.4 KE3: EE2 and BPA Exposure Impaired Gonadal Development in Male Embryos

EDCs can impair estrogen homeostasis and have many negative effects during embryonic development. Early life stage exposure to EDCs can have long-term effects on reproduction.^40^ Gonadal development in birds is mediated by the ER,^41^ and thus, evaluating alterations in gonadal structure provides a means to phenotypically anchor (at the tissue level) exposure to estrogenic chemicals. Male embryos (confirmed via genetic sex determination) exposed via egg injection prior to incubation to DMSO, EE2 (0.5, 25 μg/g), and BPA (50 μg/g) were included for gonad histology analysis at ED20. Female embryos were excluded because there were no ED20 females in the EE2 25 μg/g group. The DMSO-treated control males had normal testicular development with thin capsules and organized seminiferous tubules (Figure 4A). Exposure to the lowest dose of EE2 (0.5 μg/g) resulted in a thin layer of fibroblasts, the absence of testicular cords, and a thick layer of disorganized oocyte-type germ cells containing some strands of fibroblasts. These changes are interpreted as ovotestis (Figure 4B). There was a complete absence of testicular cords, and the outer layer consisted of dense populations of oocyte type germ cells, which can be interpreted as ovotestis following exposure to 25 μg/g of EE2 (Figure 4C). Exposure to 50 μg/g BPA resulted in thin connective tissue and loss of cord density, leading to more interstitial tissue replacing the functional germ cells. The stroma appeared more aqueous, and there was a clear loss of seminiferous tubules compared to the DMSO control (Figure 4D). Similar to the present study, Japanese quail and chicken embryos exposed to EE2 or BPA had thick cortex and oocyte-like cells in the left testis.^9,42^ In this study, exposure to EE2 (25 μg/g) reduced the gonad weight in genetically male ED20 embryos (Figure S1). Exposure to EE2 and BPA during gonadal differentiation skewed the sex ratio toward females in fish.^43^ The histological findings indicate that both EE2 and BPA impaired testis development in chicken embryos but the effect was more pronounced after EE2 exposure. Such developmental effects on gonadal tissue have been associated with decreased fecundity/reproductive success in birds.^40^

**Figure 4:**
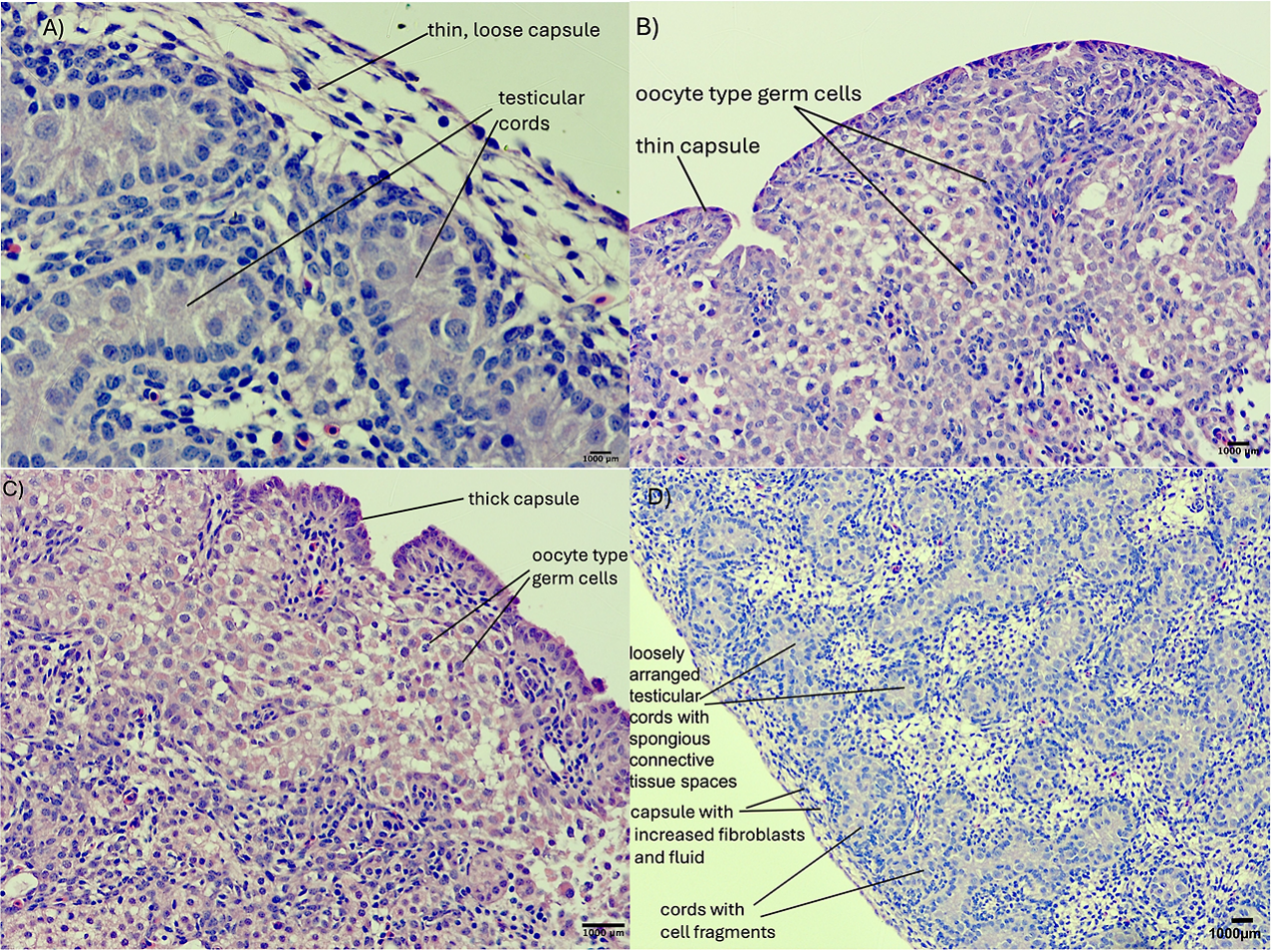
Effect of in ovo exposure to (A) DMSO, (B) EE2 (0.5 μg/g), (C) EE2 (25 μg/g), and (D) BPA (50 μg/g) on testis development in male chicken embryos at ED20.

### 3.5 EE2 and BPA Altered Morphometric Parameters and Embryonic Viability

Exposure to EE2 (50 μg/g) significantly decreased embryo mass (*p* ≤ 0.05), and 25 and 50 μg/g significantly decreased tarsus length (*p* ≤ 0.05) compared to the DMSO control (Figure 5). There were no treatment-related effects in any of the dose groups for BPA (Figure S2). Zebrafish embryos exposed to EE2 had decreased embryo mass and reduced larvae length.^44^

**Figure 5:**
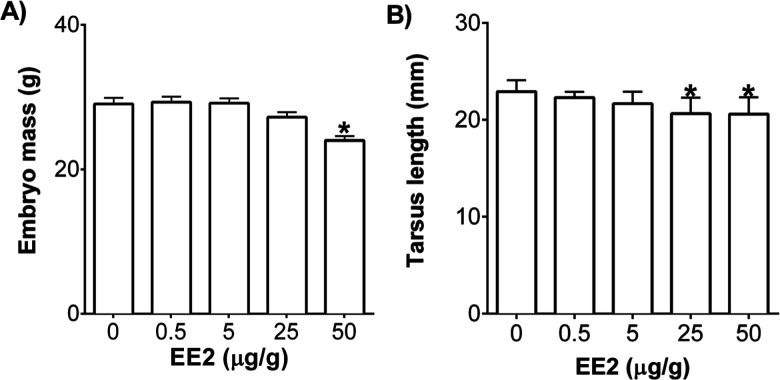
Effect of 17α-ethinylestradiol (EE2) on (A) embryo mass and (B) tarsus length at ED20 (*n* = 4–10/dose group). Error bars represent standard error of the mean (+SEM), and “*” denotes significance compared to DMSO controls (*p* ≤ 0.05).

The highest doses of EE2 (50 μg/g) and BPA (100 μg/g) reduced embryonic viability by 44 and 55%, respectively. The estimated LD_50_ values for EE2 and BPA at midincubation were 71.93 and 100.13 μg/g, respectively (Figure 6). Embryonic viability for individuals that survived to termination (ED20) after midincubation subsampling from each group was 100% for DMSO and all doses of EE2 and BPA, indicating that early stages of development were more vulnerable to the embryotoxic effects of the two chemicals. In another study, EE2 (20 ng/g egg) and BPA (75 μg/g egg) reduced chicken embryo viability by 23 and 30%, respectively.^42^ The differences in LD_50_ values could be attributed to different routes of exposure, and the embryonic day LD_50_ was determined. Jessl et al. (2018) injected the chemicals directly into the yolk on ED1 and calculated LD_50_ on ED19, whereas the chemicals were injected into the air cell and LD_50_ calculated on ED11 in the present study. Japanese quail embryos had limited reduction in viability up to 54.2 μg/g EE2 (slightly lower than the LD_50_ value in chickens from the present study),^45^ whereas BPA exposure via yolk injection reduced viability by 15 and 43% at 67 and 200 μg/g, respectively.^9^ The difference in the LD_50_ values for BPA between the two groups could be due to the egg injection methods (yolk vs air sac) and the solvents used to dissolve BPA (propylene glycol vs DMSO). The different routes of exposure and solvents could affect solubility and chemical uptake.

**Figure 6:**
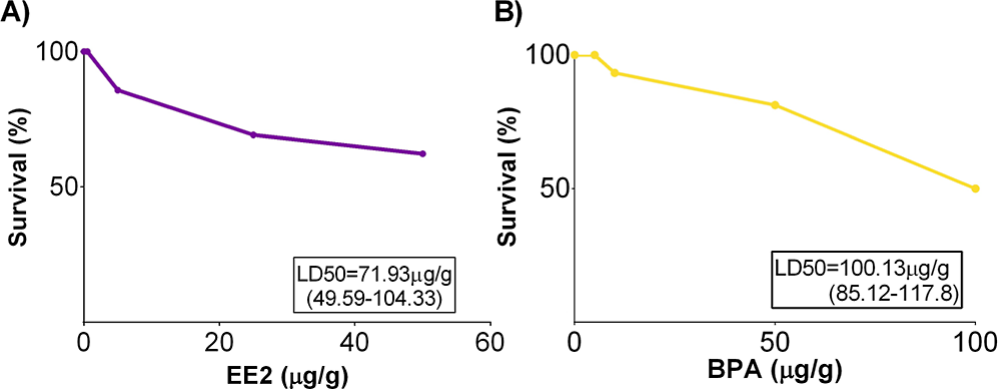
Effect of (A) 17α-ethinylestradiol (EE2) and (B) bisphenol A (BPA) on embryonic viability at mid-incubation (ED11; *n* = 14–19).

Taken together, these data contribute to an AOP (AOP 537) for an avian species, which empirically links ESR1 upregulation to modulation of estrogen-responsive genes, an increase in vitellogenesis and abnormal gonadal development, growth, and survival (Figure 7) using an early life stage model and an immortalized hepatic cell line, LMH. The early life stage model permitted evaluation of key events at different levels of biological organization, and LMH spheroids allowed for the determination of dose-dependent and temporal changes in gene expression and VTG concentrations without the requirement of animal utilization. Furthermore, the data generated using the two models support the order of the KEs and provide linkages (i.e., KERs) between KEs. There is extensive knowledge about the mechanism(s) of increased ESR1 expression leading to an increase in vitellogenesis.^46^ The MIE, ESR1 upregulation to KEs (upregulation of estrogen-responsive genes increased plasma VTG and abnormal gonad development) following exposure to estrogenic compounds during development is well-supported with empirical evidence in birds and other species such as fish.^6,47^ Evidence regarding the adverse outcome, reduced cumulative fecundity, and spawning in birds following EE2 and BPA exposure is unavailable, and long-term, multigenerational studies are needed to understand the effect of abnormal gonadal development in male and female embryos on reduced fecundity in adult birds. Exposure to EE2 led to reduced reproductive behavior and fecundity in male brackish medaka,^48^ and in another study, F0 exposure resulted in reduced fecundity in F1 male marine medaka.^49^ In utero BPA exposure (0.5 μg/kg/day) in F1 generation decreased the fertility rate in F2 and F3 generations in mice due to ovotoxicity.^50^ In female zebrafish, exposure to EE2 and BPA disrupted follicular formation,^51,52^ which was similar to the histological observations in this study (data not shown due to small sample size). Therefore, there is evidence to suggest that long-term chronic exposure to estrogenic chemicals can impact reproductive success (e.g., fecundity), and these are preceded by many of the alterations we observed at other levels of biological organization in an avian model species.

**Figure 7:**
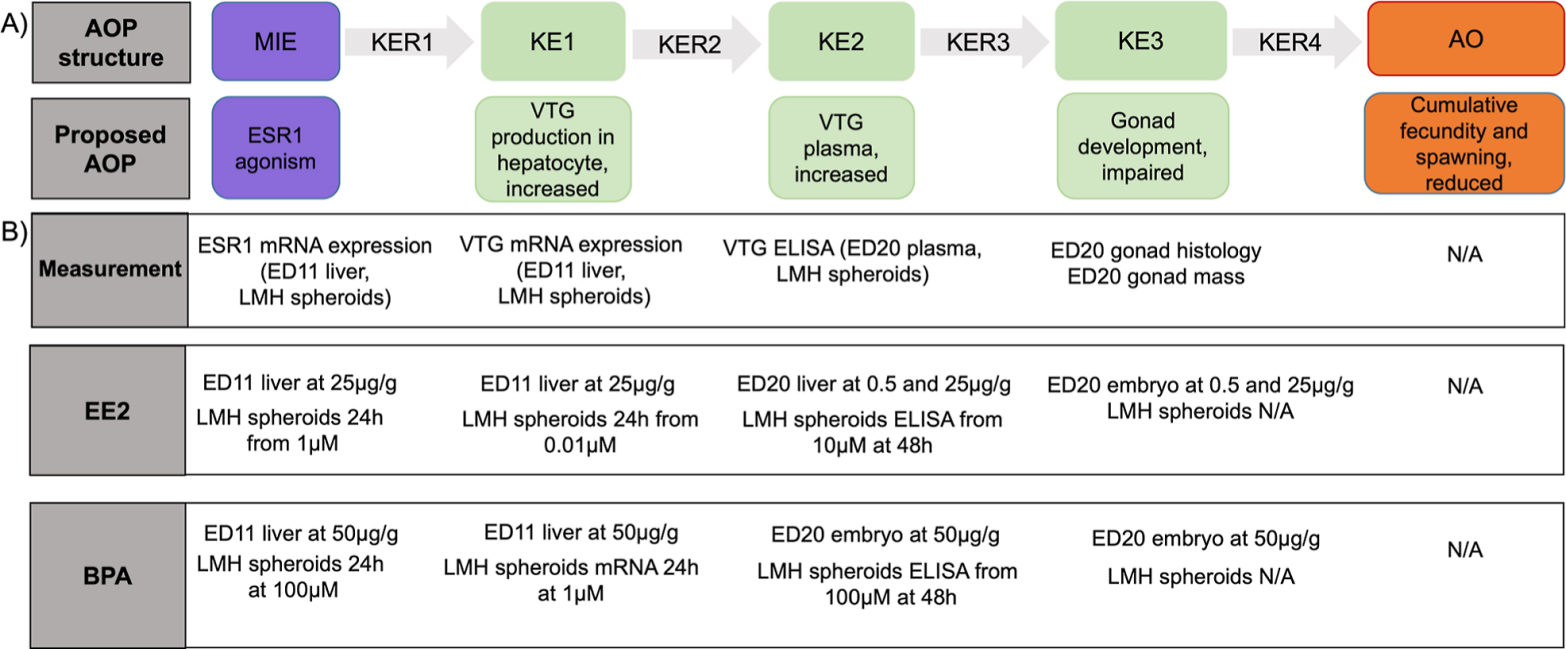
Adverse outcome pathway of estrogen receptor (ESR1) agonism leading to reduced cumulative fecundity and spawning (A) that includes the empirically derived end points measured in the present study (B; AOP537).

## Supplementary Material



## Notes

List of estrogen-responsive genes on the array; effect of EE2 on gonad weight; and effect of BPA on embryo mass, head plus bill length, and tarsus length (PDF)
