# Supplementary material for: Avian-Specific Evidence for an Estrogen Receptor Agonism Adverse Outcome Pathway Based on Chicken Embryos and LMH 3D Spheroids Exposed to Ethinylestradiol and Bisphenol A
Source: Environ Sci Technol. 2025 May 19;59(21):10136–44. doi: 10.1021/acs.est.4c10887 (PMC12138968; doi:10.1021/acs.est.4c10887)
Supplement: Supplementary file 1 [file es4c10887_si_001.pdf]

Supporting Information

Avian-specific evidence for an estrogen receptor agonism adverse outcome pathway based on chicken embryos and LMH 3D spheroids exposed to ethinylestradiol and bisphenol A

Tasnia Sharin[1], Kim L. Williams[1], Rudolf W. Mueller[2], Doug Crump[1*], Jason M. O'Brien[1]

[1]National Wildlife Research Centre, Environment and Climate Change Canada, Ottawa, Ontario, Canada

[2] Department of Pathology and Laboratory Medicine, University of Ottawa, Ottawa, Ontario, Canada

[*]Corresponding author email: doug.crump@ec.gc.ca

Summary: 4 pages, 2 figures, 1 table

Table S1. Estrogen-responsive genes on the customized array.

Pathway	Description	Symbol	Ref_Seq
Estrogen-responsive	Apovitellenin-1	APOV1	NM_205483
	Carnitine O-palmitoyltransferase 1	CPT1A	NM_001012898
	Cathepsin D-like	CTSD	NM_205177
	Cholesterol 7-alpha-monooxygenase	CYP7A1	NM_001001753
	Estrogen receptor 1	ESR1	NM_205183
	Fibroblast growth factor 19	FGF19	NM_204674
	Stearoyl-CoA desaturase	SCD	NM_204890
	Thyroid hormone responsive	THRSP	NM_213577
	Vitellogenin-2	VTG2	NM_001031276
Reference genes	Eukaryotic translation elongation factor 1 alpha 1	EEF1A1	NM_204157
	Beta-actin	β-ACT	XM_424240

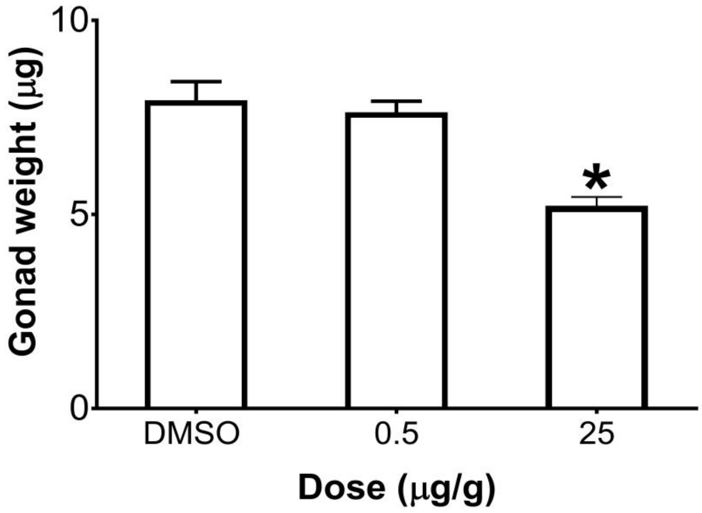

Fig S1. Effect of EE2 on gonad weight in genetically male ED20 embryos (n=3-6/dose group). Error bars represent standard of error of the mean (SEM) and "*" represents significant difference compared to DMSO-treated embryos ($p<0.05$).

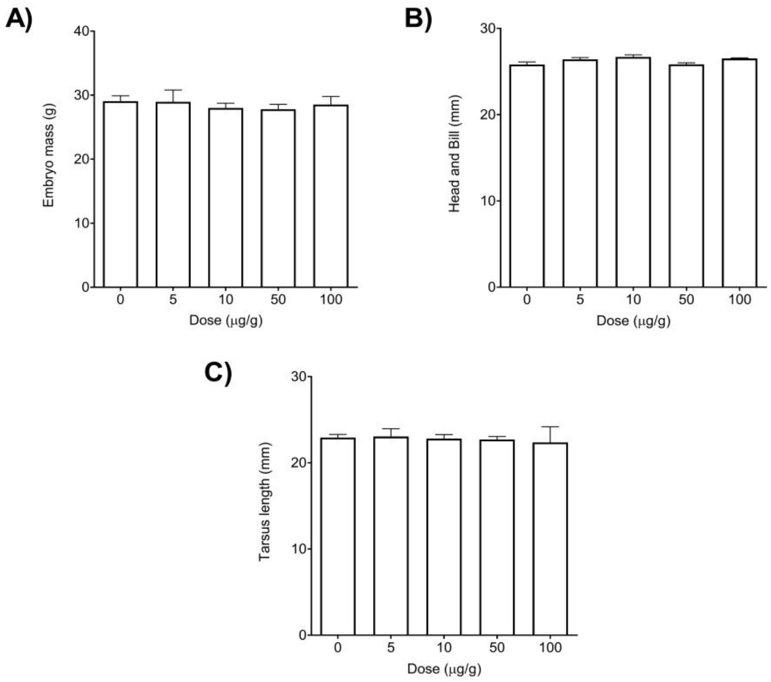

Fig S2. Effect of BPA on A) embryo mass, B) head plus bill length, and C) tarsus length at ED20 (n=5-9/dose group). Error bars represent standard of error of the mean (+SEM).
